# Renal allograft surveillance with allospecific T-cytotoxic memory cells

**DOI:** 10.1080/0886022X.2020.1846054

**Published:** 2020-11-18

**Authors:** Vinayak S. Rohan, Karim M. Soliman, Ahmad Alqassieh, Duaa Alkhader, Neha Patel, Satish N. Nadig

**Affiliations:** a Department of Surgery, Division of Transplant Surgery, Medical University of South Carolina, Charleston, SC, USA; b Department of Medicine, Division of Nephrology, Medical University of South Carolina, Charleston, SC, USA; c Department of Pharmacy, Medical University of South Carolina, Charleston, SC, USA

**Keywords:** Kidney transplantation, rejection, immune monitoring, T- cytotoxic Memory cells

## Abstract

**Background:**

Allo-antigen-specific T-cytotoxic memory cells (TcM) which express CD40 ligand (CD154) in overnight lymphocyte co-culture are strongly associated with acute cellular rejection (ACR) seen in “for cause” biopsies for renal allograft dysfunction. Specifically, when the likelihood of rejection is increased, donor-specific allospecific TcM exceed those induced by HLA-non-identical third-party cell by 1.15-fold or greater.

**Methods:**

The performance of allospecific TcM was evaluated retrospectively in primary renal transplant recipients (RTR) at routine clinical visits, cross-sectionally at presentation for biopsies, and serially. Performance metrics were sensitivity, specificity, positive and negative predictive values (PPV and NPV).

**Results:**

Twenty-two primary RTR, median age 45 years (range 19–72) were tested with allospecific CD154 + TcM. Samples were obtained at the mean ± SD time interval of 806 ± 239 days after kidney transplantation. Six of 22 patients experienced biopsy proven T- Cell Mediated Rejection (TCMR). A seventh showed antibody mediated rejection (ABMR). Of these seven patients six demonstrated increased likelihood of rejection with allospecific TcM (sensitivity 83%). Ten of these 15 patients with no rejection had a negative test (specificity 67%). False positive tests were seen in five patients. Six out of 11 patients with positive tests had ACR/ABMR with a PPV of 54%, while 10 out of 11 patients with negative tests were non-rejecters with a NPV of 91%.

**Conclusion:**

Allospecific T-cytotoxic memory cells distinguished primary RTR with quiescent allografts from those with dysfunction. With serial surveillance measures, this test system may facilitate decisions to manage immunosuppression in RTR.

## Introduction

Determining the risk of renal transplant rejection can lead to early diagnosis and intervention and enhance graft survival. Previous studies have shown that enhanced donor-specific alloreactivity measured with allospecific CD154-expressing T-cytotoxic memory cells (CD154 + TcM) predicts ACR after liver or intestine transplantation in children [1–3]. This FDA-approved test has a sensitivity and specificity approaching or exceeding 80% [3]. In an exploratory cohort of patients with metabolic liver disease who were treated with hepatocyte transplantation, loss of graft function was preceded by an increase in donor-specific CD154 + TcM [4]. In another cohort of liver transplant recipients, infusion of T-regulatory cells resulted in reduced the frequencies of circulating donor-specific CD154 + TcM [5]. Finally, intestine allografts with ACR, which is usually accompanied by circulating DSA were associated with enhanced donor-specific alloreactivity measured with CD154 expression in both, the TcM and the B-cell compartments [6]. Among RTR undergoing ‘for cause’ biopsies for elevated serum creatinine (SCr), allospecific CD154 + TcM demonstrated sensitivity and specificity exceeding 80% for association with T-cell-mediated rejection (TCMR) [7]. It is not known whether allospecific TcM can also predict outcomes in a mixed cohort of RTR with stable graft function, or graft dysfunction due to various causes, as is typically seen in a clinical practice.

Since the availability as a clinical test, allospecific CD154 + TcM have been used cross-sectionally and serially in primary RTR who are stable or experiencing allograft dysfunction at our center. Here we report test performance in the first 22 RTR. Samples were drawn at the time of biopsy either for graft dysfunction (majority of the patients) or during routine follow up biopsies after treatment for borderline rejection. Specifically, we asked whether donor-specific alloreactivity measured with CD154 + TcM were associated with rejection, graft dysfunction and immunosuppression.

## Materials AND methods

All data were collected and analyzed after approval by the Medical University of South Carolina’s Institutional Review Board (IRB ID-Pro00098072). Patients younger than <18 years of age, multiorgan transplants were excluded from the study. Blood samples, 5-10 mL, from 22 adult RTR were obtained serially during routine clinical visits and/or cross-sectionally at the time of biopsy before any treatment for rejection was instituted. Serial samples were taken at 3-4 week intervals. Blood samples were collected in sodium heparin tubes and shipped overnight at ambient temperature to the reference laboratory for rejection risk assessment (Pleximark^TM^, Plexision Inc., Pittsburgh, PA 15224). Frequencies of CD154 + TcM induced by overnight stimulation with donor-HLA-matched (donor) PBL were measured with flow cytometry. Donor-specific CD154 + TcM were expressed as a multiple of those induced by stimulation with HLA-mismatched PBL in parallel co-culture, as described previously. This multiple was reported as an index of rejection (IR). An IR of 1.15 or greater implied increased likelihood of rejection. This threshold was identified previously from training set-validation set testing of 43 RTR sampled at the time of graft biopsies for renal dysfunction [7]. Performance metrics were sensitivity, specificity, PPV and NPV values.

## Results

### Human subjects

Twenty-two primary RTR, median age 45 years (range 19–72) were tested with allospecific CD154 + TcM. The distribution of male: female gender was 16:6; African American: Caucasian: Hispanic races was 13:8:1; and deceased donor: living donor: donations after cardiac death (DCD) graft was 12:7:3. In all patients, immunosuppression on the day of sampling consisted of triple maintenance therapy of oral tacrolimus, mycophenolate mofetil and corticosteroids. Samples were obtained at the mean ± SD time interval of 806 ± 239 days after renal transplantation.

### Samples

From the 22 RTR, a total of 26 samples were tested. Samples selected for analysis included single cross-sectional samples from 18 RTR, and the first of two serial samples from each of four RTR. The results of each sample are shown in Table 1. The distribution of demographics and results of 11 samples denoted increased likelihood of rejection while the other 11 samples denoted decreased likelihood of rejection (Table 2).

**Table 1.** Outcome of each kidney transplant recipient undergoing the allospecific CD154 + TcM test.

Series No	Sample type	Donor type	Age at sampling	IR	Test interpretation: likelihood for rejection	Test Performance	Clinical/biopsy decision basis	Tacrolimus ng/mL	Creatinine mg/dL
1	Cross-sectional	Living	41.17	0.712	Not likely	True Negative	Clinical	24.4	1.48
2	Cross-sectional	Cadaveric	58.03	0.667	Not likely	True negative	Biopsy	9.8	2.00
3	Cross-sectional	Cadaveric	30.98	1.066	Not likely	True negative	Biopsy	8	1.79
4	Cross-sectional	Cadaveric	29.32	1.140	Not likely	True negative	Clinical	22.6	1.20
5	cross-sectional	Cadaveric	61.24	0.494	Not likely	True negative	Biopsy	7.1	1.30
6	Cross-sectional	Cadaveric	72.42	1.034	Not likely	True negative	Biopsy	11.4	0.80
7	Cross-sectional	Cadaveric	47.37	0.579	Not likely	True negative	Biopsy	8.9	1.50
8	Cross-sectional	Cadaveric	61.72	0.600	Not likely	True negative	Biopsy	10.5	1.10
9	Cross-sectional	Living	22.86	0.533	Not likely	True negative	Biopsy	8.2	2.10
10	Cross-sectional	Living	19.25	0.578	Not likely	True negative	Biopsy		3.50
11	First of all serial samples	Cadaveric	33.42	0.750	Not likely	False negative	Biopsy	15.5	1.90
12	Cross-sectional	Living	61.65	1.968	Likely	False positive- ATN	Biopsy	8.5	1.50
13	Cross-sectional	DCD	64.18	1.485	Likely	False positive-BK	Biopsy	11	2.50
14	Cross-sectional	DCD	53.57	2.205	Likely	False positive	Biopsy	7.5	3.10
15	Cross-sectional	DCD	39.16	1.311	Likely	False positive-BK	Biopsy	12.5	2.30
16	First of all serial samples	Living	44.86	1.167	Likely	False positive	Biopsy	7.7	1.70
17	Cross-sectional	Living	49.69	2.000	Likely	ABMR DSA-MFI 1364 class II	Biopsy	9.2	4.80
18	First of all serial samples	Cadaveric	44.46	1.245	Likely	True positive	Biopsy	12.2	1.30
19	First of all serial samples	Cadaveric	34.60	2.235	Likely	True positive	Biopsy	2.7	3.30
20	Cross-sectional	Cadaveric	30.44	1.370	Likely	True positive	Biopsy	2.9	2.30
21	Cross-sectional	Living	63.99	1.714	Likely	True positive	Biopsy	4.5	1.30
22	Cross-sectional	Cadaveric	35.51	1.229	Likely	True positive	Biopsy	11	1.40

ATN: Acute Tubular Necrosis; BK: BK virus nephropathy; DCD: Donations after cardiac death; ABMR: Antibody mediated rejection; DSA: Donor specific antibody; MFI: Mean fluorescence intensity.

**Table 2.** Demographics and outcomes in samples associated with increased and decreased likelihood of rejection (mean ± SEM) using t-test.

Assay outcomes	Not likely rejection	Likely rejection	*p* Value Two tail
Number	11	11	
Male: Female	7:04	9:02	
Caucasians: African American: Other	4:06:01	4:07:00	
Donors: Cadaveric :Living related: Donations after cardiac death	8:03:00	4:04:03	
Age at sample collection (years)			
Mean ± SEM	43 ± 5	47 ± 4	0.54
Range	19 to72	30 to 64	
Time from Transplant to sample (days)			
Mean ± SEM	1044 ± 345	568 ± 332	0.33
Range	26 to3481	19 to 3752	
Glomerular Filtration Rate, at the time of sample (mL/min/ 1.73 m^2^)			
Mean ± SEM	50 ± 4	41 ± 5	0.22
Range	24 to 60	15 to 69	
Creatinine level at the time of sample (mg/dL)			
Mean ± SEM	1.7 ± 0.2	2.3 ± 0.3	0.13
Range	0.8 to 3.5	1.3 to 4.8	
Tacrolimus level at the time of sample (ng/mL)			
Mean ± SEM	12.6 ± 2.0	8.2 ± 1.1	**0.06**
Range	7.1 to 24.4	2.7 to 12.5	

Bold values represents as significant values.

### Allospecific CD154 + TcM associated with rejection after RTR

**Figure 1.**
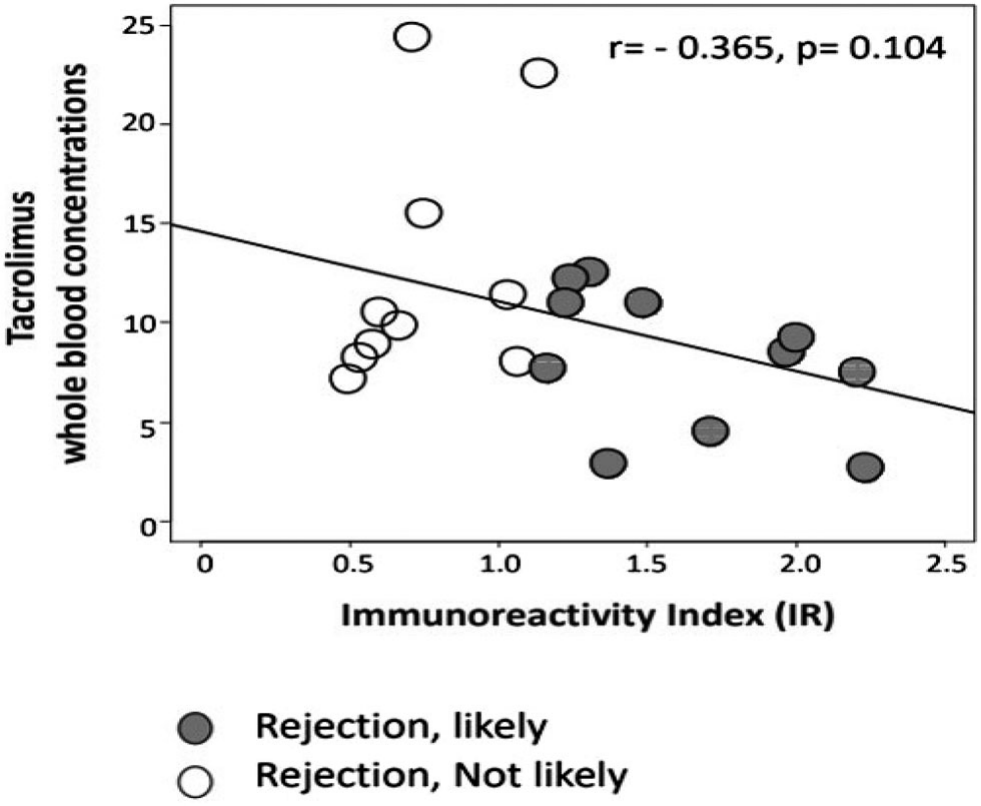
The numeric IR values inversely correlated with tacrolimus whole blood concentrations, suggestive but not statistically significant (*r* = −0.365, *p* = 0.104).

**Figure 2.**
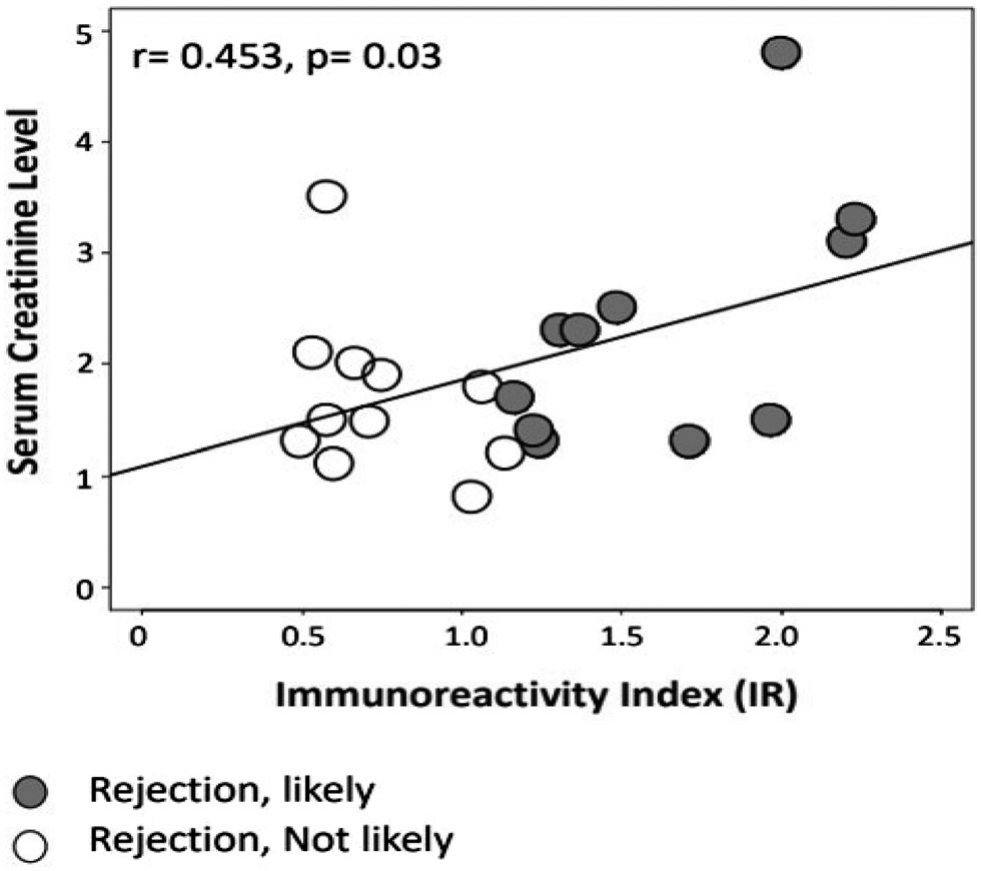
The numeric IR values positively correlated with SCr levels, statistically significant (*r* = 0.453, *p* = 0.03, Pearson test).

Six of 22 patients experienced biopsy proven TCMR. A seventh showed ABMR at biopsy for allograft dysfunction and circulating donor-specific anti-HLA antibodies (DSA). Of these seven patients with ACR or ABMR, six demonstrated increased likelihood of rejection with allospecific TcM or a positive test (sensitivity 83%). Of the remaining 15 patients, 12 had stable SCr consistent with absence of ACR, while biopsies in the remaining three showed acute tubular necrosis (ATN) in one and stage II and III BK nephropathy in two DCD RTR allografts. Ten of these 15 patients with no rejection had a negative test or decreased likelihood of rejection (specificity 67%). False positive tests were seen in five patients, one of whom had no rejection. In the remaining four patients, one had ATN, and three were recipients of DCD allografts, of whom two had BK nephropathy. Of 11 patients with positive tests, 6 had ACR/ABMR with a PPV of 54%. Of 11 patients with negative tests, 10 were non-rejecters with NPV of 91%. By using the two-tailed (t-test) correlation, we first compared SCr levels in RTR with “rejection-likely” and “rejection-not-likely” signals (Table 2). An increasing IR values of CD154 + TcM were suggestive but not reaching a statistical significant correlation with decrease in tacrolimus whole blood concentrations (r = −0.365, *p* = 0.104) (Figure 1). Then we explored the dynamic range of SCr levels with dynamic ranges of IR values using Pearson’s correlation, the latter being considered a more sensitive analysis than the t-test. The p value was 0.03 (*r* = 0.453), demonstrating statistical significance (Figure 2). These findings suggest that the borderline significance we saw in the two-group comparison with a t-test is meaningful in this modest cohort.

### Allospecific CD154 + TcM associated with graft dysfunction

Between-group comparisons showed that SCr was higher (Mean ± SEM; 2.3 ± 0.3 vs. 1.7 ± 0.2 mg/dl, *p* = 0.13) and whole blood tacrolimus concentrations were lower (Mean ± SEM; 8.2 ± 1.1 vs. 12.6 ± 2.0 ng/ml, *p* = 0.06) in 11 patients with positive tests compared with 11 patients with negative tests. The numeric IR values, SCr, and tacrolimus whole blood concentrations in 22 RTR did not follow normal distribution.

## Discussion

Our early experience showed that an IR exceeding 1.15 for allospecific CD154 + TcM was associated with histological TCMR or ABMR in RTR with a test sensitivity of 83% and a NPV of 91%. These results are remarkable and can be used to monitor the likelihood of rejection during routine minimization of immunosuppression. Downward titration accompanied by a negative test may thus disfavor rejection, while an unchecked increase in donor-specific cellular alloreactivity during this process may precipitate subclinical injury by recruiting humoral or other mechanisms of allograft injury. This likelihood of injury with suboptimal immunosuppression is suggested by our findings that demonstrated the association of increasing IR values of CD154 + TcM with increasing SCr levels and decrease in tacrolimus whole blood concentrations.

False-positive tests expand on these observations but also suggest that other explanations must be considered. Three of five such tests were performed relatively early, between days 19-121 after transplantation, and were associated with lower ranges of therapeutic tacrolimus whole blood concentrations of 7.5–8.5 ng/ml. One of these patients had ongoing ATN. The remaining two patients with tacrolimus levels of 12.5 and 11 ng/ml, respectively, were found to have histological evidence of BK nephropathy, 56 and 483 days after transplantation. Whether enhanced donor-specific alloreactivity was augmented by virus-induced inflammation, or enhanced secondary to cross-reactive antiviral immune response are possibilities that can only be addressed with sequential monitoring performed before, during and after any such episode [8, 9].

Allospecific CD154 + TcM have been successfully used in monitoring rejection in liver and intestinal transplant recipients [5,6,10,11]. Renal transplantation adds to the complexities in monitoring due to pathologies specific to RTR like ATN and BK nephropathy which cause modulation in T cell responses, and also precipitate reduction in immunosuppression, both of which might possibly lead to false positivity. A previous study by Ashokkumar et al. utilizing allospecific CD154 + TcM in 43 RTR showed a sensitivity and specificity of 88% in identifying rejection with IR 1.15 or greater. Interestingly in their study, IR of allospecific CD154 + TcM increased significantly with increasing histological severity of ACR, whether borderline, Banff 1 A, or IB [7]. In our study, increased alloreactivity was associated with graft dysfunction and lower tacrolimus levels, both findings may indicate rejection. Thus, longitudinal tracking of patients with serial measurements after treatment of rejection may allow avoidance of multiple biopsies.

The present study has a few limitations. It is a single center study with a limited number of patients. Most of the patients in the study were monitored with a single cross-sectional test. The number of patients with BK nephropathy were small. Serial testing of more patients with BK nephropathy will help us better understand the false positivity. A larger longitudinal experience with allospecific CD154 + TcM may help us understand the long-term implications of monitoring donor-specific T-cell alloreactivity. At the same time, advantages of our single-center experience are a uniform immunosuppression protocol and monitoring by the same clinical team.

Our early experience with allospecific CD154 + TcM is encouraging. A high NPV of 91% gives confidence that patients can be monitored serially, especially after treatment for rejection, at the time of infection and changes of immunosuppression without resorting to repeated biopsies. A serial monitoring schedule can be timed to coincide with periods of risk for rejection and infection, when immunosuppressive drugs undergo major schedule changes such as the elimination or dose reduction. Implementing these principles will better define the rationale for this test system in our practice.

## Ethical approval

This material has not been published previously, in whole or part, and is not under consideration for publication elsewhere, except being presented as an oral presentation at the American Transplant Congress (ATC, May, 2020).(https://atcmeetingabstracts.com/abstract/renal-allograft-surveillance-with-allospecific-t-cytotoxic-memory-cells-which-express-cd154) This paper has no tables or figures that would require permission to reprint. The authors have no conflict of interest to declare. All authors had participated in the preparation of this manuscript, fulfilled criteria for authorship and have approved the paper in the current format. The study conforms to the ethical guidelines of the 1975 Declaration of Helsinki as reflected in the prior approval by the institution’s human research committee. This study was not supported by any grant.
